# Supplementary material for: Barriers and facilitators to pharmacovigilance activities in Pakistan: A healthcare professionals-based survey
Source: PLoS One. 2022 Jul 29;17(7):e0271587. doi: 10.1371/journal.pone.0271587 (PMC9337632; doi:10.1371/journal.pone.0271587)
Supplement: S1 Questionnaire — (DOCX) [file pone.0271587.s001.docx]

**Barriers and Facilitators towards Adverse Drug Reaction reporting system in Lahore, Pakistan**

**TICK [√] APPROPRIATE BOX**

**1.0. Demographic data**

**1.** Gender □ Male □Female

**2.** What is your age? ___________years

**3.** Your highest qualification: □ Bachelors (______________)

□ Masters / M.Phil (_________________)

□ Specialization (_________________)

□ Other (please specify) (_________________)

**4.** From which medical college/ University the first degree was obtained: ________________________________________

**5.** For how many years have you been practicing in a hospital setting? ___________________

**6.** Do you practice part time:

□ Yes

□ No

**7.** Average number of patients/ prescription seen by you per day? ____________

**For Researcher Use Only**

Respondent ID: _________

Date Received: _________

**1: Barriers of Adverse Drug Reaction Reporting**

We are interested in learning about the barriers of Adverse drug reaction reporting in a hospital setting.

Please indicate the degree, to which you disagree/agree with each of the following barriers below by circling a number from 1 to 3, where:

**1 = disagree, 2 = neutral, 3 = agree**

| **Sr** | **Barriers to ADR reporting** | **1** | **2** | **3** |
| --- | --- | --- | --- | --- |
|  | Lack of knowledge if an ADR happened |  |  |  |
|  | Lack of time for reporting |  |  |  |
|  | Lack of interest to report about an ADR |  |  |  |
|  | Lack of confidence in discussing the ADRs with the prescriber |  |  |  |
|  | The ADR form is too difficult to fill |  |  |  |
|  | Unaware of the existence of a national ADR reporting system |  |  |  |
|  | Did not know how to report an ADR in my work place |  |  |  |
|  | Fear of legal liability |  |  |  |
|  | Unaware of the need to report an ADR |  |  |  |
|  | Lack of financial reimbursement |  |  |  |
|  | Lack of support from colleagues and administration |  |  |  |
|  | Difficulties to report an ADR when patients are treated with several drugs |  |  |  |
|  | I never get back any feedback on what action is taken from pharmacovigilance centre |  |  |  |

**2: Facilitators**

We are interested to know about the possible methods for the improvement of ADR reporting Please indicate the degree, to which you disagree/agree with each of the following barriers below by circling a number from 1 to 3, where:

**1 = disagree, 2 = neutral, 3 = agree**

Please tick the appropriate answer

| **Sr.** | **Which of the following suggestion(s) would you choose to improve ADR reporting?** | **1** | **2** | **3** |
| --- | --- | --- | --- | --- |
|  | Extra time should be given to report ADRs (other than duty hours) |  |  |  |
|  | Incentives |  |  |  |
|  | Continuous medical education, training related to ADR reporting |  |  |  |
|  | Reminders and increased awareness from the ADR Monitoring Center |  |  |  |
|  | Online system for adverse drug reaction reporting should be available |  |  |  |
|  | Feedback from the drug reporting regulatory agency (DRAP) |  |  |  |
