# Supplementary material for: Barriers and facilitators to pharmacovigilance activities in Pakistan: A healthcare professionals-based survey
Source: PLoS One. 2022 Jul 29;17(7):e0271587. doi: 10.1371/journal.pone.0271587 (PMC9337632; doi:10.1371/journal.pone.0271587)
Supplement: Attachment — Submitted filename: Rebuttal Document.pdf [file pone.0271587.s005.pdf]

Rebuttals

Dear Reviewer, we would like to thank you for reviewing the manuscript and helping us to improve the quality of our manuscript.

As suggested, the manuscript has been proofread and edited by a native English speaker.

Abstract
----
1. Page 2, line 34-35: "However, barriers to the underreporting of ADRs exist in almost all healthcare systems." This statement does not sound quite right. Barriers exist causing underreporting of ADR rather than barriers to underreporting of ADR. Same in line 44-45.

Response: Many thanks, we have modified the sentence as suggested.

Line 34-35: However, in almost all healthcare systems barriers exist that lead to the underreporting of ADRs.
Line 44-45: …………………. lack of support from a colleague as a reason that could lead to the underreporting of ADR.

Introduction
----
1. Page 3, Line 61-62: Add reference for the statement "Among hospitalized patients, ADRs represent the fifth most common cause of death."

Response: Thank you. The reference has been added.

Line 63: European Commission Strengthening Pharmacovigilance to Reduce Adverse Effects of Medicines. Memo/08/782. 2008. [(accessed on 18 May 2022)]. pp. 2–5. Available online: https://ec.europa.eu/commission/presscorner/detail/de/MEMO_08_782.

2. Page 4, Line 82-83: Restructure the sentence so that the 's does not appear after the abbreviation.

Response: Many thanks, we have corrected the sentence.

Line 84-85: Pakistan joined the PIDM by the World Health Organization (WHO) as the 134th member in 2018.

3. Page 4, Line 96-97: Repeated information.

Response: Yes, repeated information has now been removed. Line 98

4. Page 5, Line 115: Repeated use of the abbreviation PV "study has analyzed the core indicators responsible for a functional PV system (PV) in Pakistan." Add reference to this statement as well.

Response: Thank you, we have removed the repeated word "PV" from the sentence and have added the reference.

Line 116: A recent study has analyzed the core indicators responsible for a functional pharmacovigilance system in Pakistan.

Reference: 19. Khan M, Hamid S, Ur-Rehman T, Babar ZJFPdf. Assessment of the Current State of PV System in Pakistan Using Indicator-Based Assessment Tool. 2022.

5. Page 5, Line 119: What is the difference between the barriers and facilitators affecting the ADR reporting system and barriers and facilitators to improve the underreporting of ADRs?

Response: Many thanks for pointing it out. We have edited the sentence for clarity.

Line 119: there is a scarcity of literature about barriers causing underreporting of ADRs and facilitators to improve the reporting of ADRs among healthcare professionals in the country.

Methods

----

1. Page 6, Line 114: Repeated use of the abbreviation HCP.

Response: Thanks, we have removed the repeated word HCP.

Line 145: The questions included demographic details and experiences of HCPs regarding barriers and facilitators toward ADR reporting in their work setting.
