# Supplementary material for: Barriers and facilitators to pharmacovigilance activities in Pakistan: A healthcare professionals-based survey
Source: PLoS One. 2022 Jul 29;17(7):e0271587. doi: 10.1371/journal.pone.0271587 (PMC9337632; doi:10.1371/journal.pone.0271587)
Supplement: Attachment — Submitted filename: Rebuttals .pdf [file pone.0271587.s006.pdf]

Abstract

1. Page 2, Line 41: “Over sixty-two percent of physicians…” change to “Over 62% of physicians…”

Response: Thank you, the suggestion is accepted. Line 41

2. Page 2, Line 43: “Similarly, 41.6% of 44 nurses identified a lack of financial reimbursement…” similar to? Unclear use of adverb.

Response: Many thanks, we have replaced “similarly” with “furthermore”. Line 44

Introduction

1. Page 4, line 95: Abbreviate Drug Regulatory Authority of Pakistan as (DRAP) at first mention.

Response: Noted and abbreviations are made standardized throughout the manuscript. Line 96

2. Page 5, line 106: Already abbreviated in line 88. Since it is the beginning of the sentence, the full form can be used without stating the abbreviation.

Response: Many thanks and we have made the change as suggested. Line 107

3. Page 5, line 106: Healthcare professionals play an important role in the establishment and “working” of pharmacovigilance in a country. Suggest to replace “working” with “functionality”.

Response: Thank you and we have replaced the word as suggested. Line 108

4. Page 5, line 107: Hence it is important to understand “the” barriers and facilitators…

Response: Thank you for pointing it out. We have added “the” as suggested. Line 108

Results

1. Page 8, Line 191: A rate has to be %, not a number. Suggest to rephrase to “The returned questionnaires were collected by the researcher with a response rate of 90.1% (n=346) for all participating HCPs.”

Response: Many thanks. We have rephrased the sentence as advised. Line 192

2. Page 9, Line 209: spacing between “and” and “17”.

Response: Thank you, space is provided between “and” and “17”. Line 210

Discussion

1. Page 10, Line 248: This “could be” evident from the studies… Change “could be” to “was”.

Response: Thank you, we have replaced “could be” with “was”. Line 249
