# Supplementary material for: Barriers and facilitators to pharmacovigilance activities in Pakistan: A healthcare professionals-based survey
Source: PLoS One. 2022 Jul 29;17(7):e0271587. doi: 10.1371/journal.pone.0271587 (PMC9337632; doi:10.1371/journal.pone.0271587)
Supplement: Attachment — Submitted filename: REVIEWER COMMENTS.docx [file pone.0271587.s003.docx]

Thank you for the opportunity to review the manuscript entitled “Barriers and facilitators to adverse drug reaction reporting by healthcare professionals: a cross-sectional survey”. The authors have conducted a questionnaire-based study to determine the barriers and facilitators related to adverse drug reaction (ADR) reporting of physicians, pharmacists and nurses in Lahore, Pakistan. Although assessing the knowledge, attitude and practice of the healthcare professionals (HCPs) in ADR reporting would have been more appropriate for a country with infancy stage of pharmacovigilance activity, the authors have identified the barriers and facilitators of the ADR reporting.

Overall, the manuscript contains many grammatical errors and would greatly benefit from proofreading and language editing.

Abstract

1. Page 2, Line 33: Abbreviate adverse drug reaction at first mention.
2. There is no mention of facilitators of ADR in the background and objective.
3. The objective mentioned in the abstract and in the main text is not the same. The objective in the abstract includes identifying possible solutions to improve the underreporting of ADRs among the healthcare professionals from Lahore, Pakistan. However, the objective in the main text does not outline this component.
4. Page 2, Line 54: Lack of knowledge or awareness (as mentioned in line 47)? Standardized use of keywords should be followed.

Introduction

1. Page 4, Line 80: The word adverse drug reaction should have been abbreviated at first mention in Line 80 if abbreviation in abstract not considered as first mention. The abbreviation and full name have been used interchangeably throughout the manuscript. Standardize the use of abbreviation (where appropriate) after first mention.
2. Page 4, Line 86 & 87: Include reference(s).
3. Page 5, line 101-102: Grammar correction “To date, there are a total of 171 member countries for the PIDM…”
4. Page 5, Line 104: PIDM already been abbreviated at first mention (line 99). Delete the full name and use abbreviation instead.
5. Include ADR prevalence data in Pakistan.
6. Include data on the knowledge, attitude and practice towards ADR reporting of HCPs in Pakistan.
7. Include evidence to establish that under-reporting of ADR is an issue in Pakistan.

Method

1. Page 6, Line 129: Healthcare or health care? Use standardized term.
2. The eligibility criteria of the respondents could be described in further details. Were trainees included? How about those who were on long leave during the data collection period?
3. Include a brief description of the study site(s).
4. Page 7, line 145: HCP was not abbreviated at first mention.
5. Include information on the reliability and validity of the questionnaire.
6. Was a pilot testing of the questionnaire conducted?
7. Include information on the distribution of the questionnaire. Was a hardcopy or softcopy version used? How much time was given for the respondents to return the completed questionnaire? What are the measures taken to increase response rate?
8. Page 7, Line 154: Under-reporting or underreporting? Use standardized term.
9. Page 7, Line 154: Delete repeated word “and and”
10. Page 7, Line 154: Spelling error “associatrion”
11. Usually (not always), the number of questionnaire distributed will be more than the calculated sample size to account for losses and refusal to participate. Why wasn’t this done?

Results

1. Page 7 & 8, Line 161 – 168: The information presented seem redundant as already summarized in Table 1.
2. As the level of knowledge of the HCPs were not assessed, the reported lack of knowledge seems to be what was perceived by the HCPs as opposed to the actual scenario. The term “perceived lack of knowledge” maybe more suitable to be used in the results, discussion and conclusion section.
3. Suggest to combine Figure 1A, 1B and 1C to present the overall results and comparison of the barriers related to ADR reporting by healthcare professional category. The same may be done to present the data on the facilitators to ADR reporting.
4. Overall, the inferential analysis data may be summarized and presented to complement the data in the Fig.3 and 4.

Discussion

1. Page 11, Line 242-244: This information should be in “Results” section.
2. Page 12, Line 267-268: Include more references to denote the plurality in the statement “Studies have identified that a lack of confidence in determining whether a drug has caused a reaction also adds to one of the determinants of under-reporting (24).”
3. Page 12, Line 273-274: What is meant by “identified a lack of reporting system as a barrier hindering the reporting of an ADR”? Lack of system or lack of awareness to system?
4. Page 12, Line 274: The word “barrier” or “hindering” should be used. Not both.
5. Page 13, Line 289-290: The lack of knowledge, skill and training was not assessed in this study making the statement “The current study has identified the lack of knowledge, skills and training of study participants, especially among physicians and nurses” invalid.
6. Page 14, Line 313: WHO was not abbreviated at first mention.
7. What are the strength and limitations of the study?

Conclusion

1. The conclusion could be rephrased as lack of knowledge was not assessed in this study.

Tables & Figure

1. The number of tables and figures can be reduced.
2. Page 29: Fig 2. Analysis of facilitators based on demographic characteristics. This should be Fig.4
